# Natural Deep Eutectic Solvents Enhanced Electro-Enzymatic Conversion of CO_2_ to Methanol

**DOI:** 10.3389/fchem.2022.894106

**Published:** 2022-05-27

**Authors:** Zhibo Zhang, Hui Wang, Yi Nie, Xiangping Zhang, Xiaoyan Ji

**Affiliations:** ^1^ Energy Engineering, Division of Energy Science, Luleå University of Technology, Luleå, Sweden; ^2^ State Key Laboratory of Materials-Oriented Chemical Engineering, Nanjing Tech University, Nanjing, China; ^3^ Zhengzhou Institute of Emerging Industrial Technology, Zhengzhou, China; ^4^ Beijing Key Laboratory of Ionic Liquids Clean Process, CAS Key Laboratory of Green Process and Engineering, State Key Laboratory of Multiphase Complex Systems, Institute of Process Engineering, Chinese Academy of Sciences, Beijing, China

**Keywords:** CO_2_ conversion, enzyme, NADESs, electrocatalysis, methanol

## Abstract

Electro-enzymatic conversion of CO_2_ offers a promising solution for CO_2_ utilization, while the conversion rate and efficiency were disappointing. To address the challenge, four kinds of natural deep eutectic solvents (NADES) with desirable biocompatibility were developed for the first time and used as the co-electrolyte in the electro-enzymatic conversion of CO_2_. As a result, the SerGly-based solution presents high CO_2_ solubility and high electrocatalytic activity, compared to the conventional buffer. By applying SerGly in the electro-enzymatic conversion of CO_2_, the yield of the product (methanol) is two times higher than that in the Tris-HCl buffer (0.22 mM) and 16 times higher than the control reaction.

## Introduction

To achieve energy sustainable development, the transformation of CO_2_ to high-valued chemicals or fuels, e.g., methanol, is an ideal strategy to both alleviate climate issues (greenhouse effect) and promote resource recycling (Behrens et al., 2012). Up to now, extensive efforts have been made devoted to the methanol synthesis from CO_2_ by using chemical, photochemical, electrochemical, and enzymatic conversion methods (Wang et al., 2011; Kondratenko et al., 2013; Zhang et al., 2021a). Due to the inherent thermodynamic stability and inertness of CO_2_, it is generally hard to convert CO_2_, typically along with high energy-demand, low efficiency, and low selectivity (Fu et al., 2020; Masood ul Hasan et al., 2021). Comparatively, enzymatic conversion of CO_2_ can be promising solutions with high efficiency, high selectivity, and mild conditions with environmental friendliness (Shi et al., 2015).

Inspired by the biological metabolic pathway, sequential enzymatic conversion of CO_2_ to methanol (CO_2_ → formic acid → formaldehyde → methanol) can be realized by formate dehydrogenase (FDH), formaldehyde dehydrogenase (FaldDH), and alcohol dehydrogenase (ADH) (Wang et al., 2014). However, one of the biggest challenges in this multi-enzymatic reaction is cofactor NADH (reduced nicotinamide adenine dinucleotide) regeneration, since NADH as a sacrificial reagent provides enzyme with hydrogen and electrons at each step of reaction and then is oxidized to NAD^+^ (oxidized nicotinamide adenine dinucleotide) (Wu et al., 2013). Besides, as this multi-enzymatic reaction is reversible, the byproduct NAD^+^ has a negative effect on the equilibrium of reaction and exhibits an inhibitory effect on methanol production (Jaworek et al., 2021; Zhang et al., 2021d). Therefore, coupling the reduction of NAD^+^ to NADH (NADH regeneration) with the enzymatic conversion is essential in which not only removes the byproduct and enhances CO_2_ conversion but also cuts down the cost significantly to make the enzymatic reaction sustainable.

To date, enzymatic regeneration, photochemical regeneration, and electrochemical regeneration of NADH have been attempted in coupling with enzymatic conversion of CO_2_ (Zhang et al., 2022). For example, Zhang et al. (2018) used glucose dehydrogenase for reducing NAD^+^ to NADH integrating enzymatic conversion of CO_2_ to methanol. Four enzymes, i.e., FDH, FaldDH, ADH, and glucose dehydrogenase, were co-immobilized in the regenerated cellulose membrane for enzymatic CO_2_ conversion with cofactor regeneration. The results showed that the yield of methanol reached 73% in 30 min and then increased to 100% after coupling glucose dehydrogenase. The enzymatic regeneration was widely studied by other works (Voges et al., 2017a; Voges et al., 2017b). Rajesh et al. (Yadav et al., 2014) developed a porphyrin-based photosensitizer for photochemical regeneration of NADH. By combining enzymatic conversion of CO_2_ to methanol, the methanol concentration with photochemical NADH regeneration was efficiently improved. Likewise, Chen et al. (2019) fabricated the Rh complex-based cathode for electrochemical reduction of NAD^+^ to NADH. By integrating with the enzymatic conversion of CO_2_ to formic acid, the formic acid was generated at a rate of 79 mM/h in a sustainable way, which is far higher than the currently reported works. Compared to the high cost of enzymatic regeneration and the unstable and complex system of photocatalytic regeneration, the electrochemical regeneration of NADH is much “cleaner” with lower cost and only electrons were consumed, being a better solution. However, the electro-enzymatic conversion of CO_2_ also suffers from low conversion rate and efficiency, where the low CO_2_ concentration and transfer rate as well as the electron transfer rate can be the reasons.

To address the above issue and improve reaction efficiency, developing a novel electrolyte, which can significantly dissolve CO_2_ and improve electrocatalytic performance through enhancing CO_2_ absorption and electron-transfer, can be a promising solution. Ionic liquids (ILs) are molten salts at room temperature with low vapour pressure, high conductivity, and wide electrochemical window, which have received much attention and been widely used in CO_2_ capture, electrocatalysis, biocatalysis, and energy storage, etc. (Zhang et al., 2021b) DESs, with much easier preparation process while sharing similar properties to ILs, have also been widely used in capturing CO_2_, reducing overpotential in the electrocatalysis, and enhancing the activity of the enzyme in biocatalysis. For example, Zeng et al. (Yan et al., 2020) synthesized superbase DES that exhibited a superior high CO_2_ absorption capacity up to 0.141 g-CO_2_/g-DES. Furthermore, Han et al. (Yang et al., 2019) fabricated copper selenide catalysts for the electroreduction of CO_2_ to methanol. Under the support from ILs-based electrolyte, the current density reaches 41.5 mA/cm^2^ with a Faradaic efficiency of 77.6%. Similarly, natural deep eutectic solvents (NADESs) were developed for biocatalysis, presenting high biocompatibility, and desirable performance. According to Yang et al. (2017), the yield of the reaction was significantly increased up to 181% by employing NADESs. All these publications demonstrated that using DESs-based electrolyte is promising to improve the CO_2_ conversion in electro-enzymatic catalysis.

In this work, DESs were proposed to be used as the electrolyte in the reaction, which was expected to remarkably improve the efficiency of electro-enzymatic conversion of CO_2_. Four NADESs, i.e., glutamate glycerol (GluGly), serine glycerol (SerGly), arginine glycerol (ArgGly), histidine glycerol (HisGly), were synthesized for the first time. Characterization as well as determination of physiochemical properties and CO_2_ capture capacity of NADESs were conducted. Besides, enzyme activity, enzymatic reaction, and electrocatalytic performance for NADH regeneration in the NADESs were investigated.

## Experimental Section

### Materials

Formate dehydrogenase from Candida boidinii (FDH, EC.1.2.1.2, homo-dimer, 76 kDa), formaldehyde dehydrogenase from Pseudomonas sp. (FaldDH, EC.1.2.1.46, homo-dimer, 150 kDa), yeast alcohol dehydrogenase (ADH, EC 1.1.1.1, 141 kDa), reduced and oxidized nicotinamide adenine dinucleotide (NADH/NAD^+^, 98 wt%), L-Histidine (99%), glycerol (99%), L-glutamic acid (99%), L-serine (99%), L-arginine (99%), dopamine hydrochloride (DA), poly (ethyleneimine) (PEI), 2,2′-bipyridyl-5,5′-dicarboxylic acid, and dichloro-(penta-methylcyclopentadienyl)rhodium (III) dimer ([Cp*RhCl_2_]_2_) were purchased from Sigma-Aldrich (St Louis, MO, United States). CO_2_ gas (>99.5%) in a cylinder was purchased from Linde Gas (Sweden).

### Synthesize of NADESs

NADESs were synthesized with the method referring to the literature (Ren et al., 2018). Taking SerGly as an example, SerGly was synthesized by mixing L-serine with glycerol at a molar ratio of 1:6, and the mixture was heated at 70°C until the liquid changed from colorless to colored. The unreacted amino acid was removed by centrifugation, and the obtained product was dried under vacuum at 60°C for 2 h. Similarly, other NADESs were synthesized following the same procedure, and the obtained NADESs were stored in a glassware dryer at ambient temperature. Finally, the yields of GluGly, SerGly, ArgGly, and HisGly were 92, 95, 100, and 89%, respectively.

### Activity Assay of FDH

The FDH activity was determined by monitoring the absorbance changes at 340 nm during the redox reactions catalyzed by the FDH at 25°C. The oxidation of formate was conducted in the conventional buffer and NADES-contained solution, respectively. The assay solution (2 ml) contains 20 μg of FDH, 100 mM sodium formate, and 1 mM NAD^+^. One unit of oxidation activity was defined as the amount of enzyme required to produce 1 μmol of NADH per minute under standard conditions.

### Electro-Enzymatic Reduction of NAD^+^ to NADH and Characterization

Electrochemical regeneration of NADH was carried out using the CHI-760e electrochemical workstation in a conventional three-electrode H-cell. The Rh complex-grafted carbon felt fabricated according to the previous work (Zhang et al., 2021c) served as the working electrode, and the platinum wire and Ag/AgCl/KCl (3 M) were used as the counter and reference electrodes, respectively. The anodic and cathodic chambers were filled with 10 ml Tris-HCl (50 mM) buffer and degassed using N_2_ to prevent the oxidation of NADH. 1 mM NAD^+^ was added to the solution and then reduced to NADH by using the Rh-grafted electrode. The progress of NAD^+^ reduction was monitored with a spectrophotometer (Shimadzu) by measuring the absorbance at 340 nm. Cyclic voltammetry (CV) measurements were recorded at a scan rate of 50 mV⋅s^−1^ and the potential range is from −1 to 0 V. The electrochemical impedance spectroscopic (EIS) was carried out at open circuit potential (OCP) within the frequency extent of 100–100,000 Hz and at an amplitude of 5 mV.

### CO_2_ Solubility and Absorption Rate

CO_2_ absorption was determined by weighting the samples (Yan et al., 2020). Typically, 5 g of NADES-based solution was added into an absorption glass tube with an inner diameter of 2.0 cm. Then, the absorption tube was partially immersed in a water bath at the desired temperature, and the standard uncertainty of temperature was ±0.1°C. After that, CO_2_ was passed into the absorption tube at a flow rate of 100 ml/min. The weight of the captured CO_2_ was obtained by the electronic balance with an accuracy of ±0.0001 g until CO_2_ absorption in the DESs reached an equilibrium.

### Enzymatic Reaction

The conversion of CO_2_ to methanol was performed in the setup of the H-cell with three-electrodes. A mixture (10 ml) of 1 mg FDH, 1 mg FaldDH, 1 mg ADH, and 1 mM NADH (7.09 mg) in the cathode was prepared and used in all enzymatic reactions. Before reaction, CO_2_ gas was bubbled through 10 ml buffer solution (50 mM Tris-HCl buffer, pH 7.4) for 30 min to achieve CO_2_ saturation in the solution. Applying for a reduction peak potential, the electro-enzymatic reaction was initiated, and the methanol concentration was determined by GC using the internal standard method.

### Analytic Methods

An Agilent 7890B gas chromatograph (GC) equipped with a flame ionization detector (250°C) and an HP-5 column (30 m × 0.25 mm, film thickness 0.25 μm) was used for analyzing the products in the liquid phase and determining the methanol concentration by using ethyl acetate as the internal standard. The NADH concentration was measured by the UV-vis spectrophotometer (UV-1280, Shimadzu). Thermogravimetric analyses (TGA) were performed using a thermal gravimetric analyzer (Netzsch, STA449 F5, Germany) and the samples were heated from 30 to 400°C at 20.0°C/min under the nitrogen atmosphere. The freezing point of each DES was determined by a differential scanning calorimeter (DSC) (Netzsch DSC 200F3, Germany), and the samples were heated from −78 to 50°C at 10.0°C/min. The density and viscosity were measured by the Anton Paar DMA 5000 density meter with an uncertainty of ±0.0005 g cm^−3^ and Anton Paar AMVn with ±0.5% precision.

## Results and Discussion

### Characterization and Physicochemical Properties of NADESs

**FIGURE 1:**
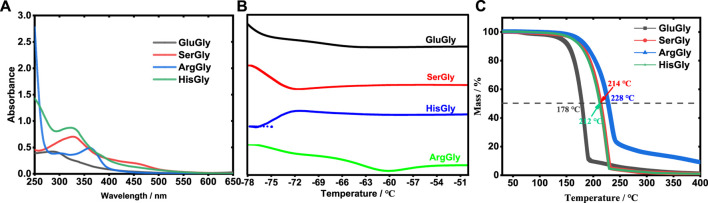
**(A)** UV curve of NADESs. **(B)** DSC curve of NADESs. **(C)** TG curve of NADESs.

In the synthesis of NADESs, it typically contains hydrogen bonding acceptor (HBA) and hydrogen bond donor (HBD), wherein the amino acid plays as hydrogen bonding acceptor (HBA), and glycerol serves as hydrogen bond donor (HBD). Taking glutamic acid as an example, the reaction between HBA and HBD was proposed in Supplementary Figure S1. To confirm the NADES formation, the synthesis process of NADESs was monitored by a UV-vis spectrometer in the wavelength ranging from 250 to 650 nm. In the beginning, the raw materials, amino acid and glycerol, do not have any absorption from 250 to 650 nm. As the reaction progressed, due to the formation of NADES, the absorption peak was observed for each NADES as shown in Figure 1A. Besides, the freezing point of NADESs was further detected by DSC. According to Figure 1B, the freezing points of GluGly (Supplementary Figure S2), SerGly, ArgGly, and HisGly were estimated to be −61, −72, −60, and −75°C, respectively, which are lower than those of amino acids (Glu: 199, Ser: 225, Arg: 260, and His: 277°C) and glycerol (17.8°C) (Do et al., 2020), also confirming the formation of NADESs successfully. The thermal stabilities of NADESs were analyzed by TG, as shown in Figure 1C. There is no obvious weight loss below 150°C for all the NADESs, indicating their high thermal stability. Also, these NADESs presented different thermal stabilities. Taking the mass loss of 50% as an example, the corresponding temperatures of GluGly, SerGly, ArgGly, and HisGly were respectively 178, 212, 214, and 228°C, and thus their thermal stabilities follow the order of ArgGly > SerGly > HisGly > GluGly. Furthermore, the obtained NADESs present super hydrophilic, which can be dissolved in water at any proportion.

**FIGURE 2:**
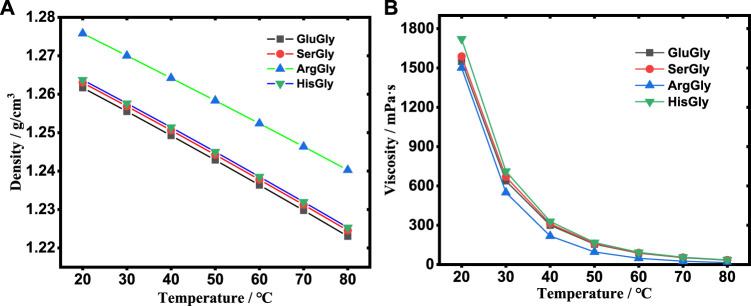
The density **(A)** and viscosity **(B)** of NADESs at different temperatures.

The density and viscosity are key properties of the reaction media. The temperature-dependent densities and viscosities were measured as shown in Figure 2 and listed in Supplementary Table S1. The results showed that the density decreases linearly with the increase of temperature for all NADESs, and follows the order of ArgGly > HisGly > SerGly > GluGly. The viscosity decreases non-linearly (sharply) with the increase of temperature for all NADESs, and follows the order of HisGly > SerGly > GluGly > ArgGly.

### Enzyme Activity in DESs

**FIGURE 3:**
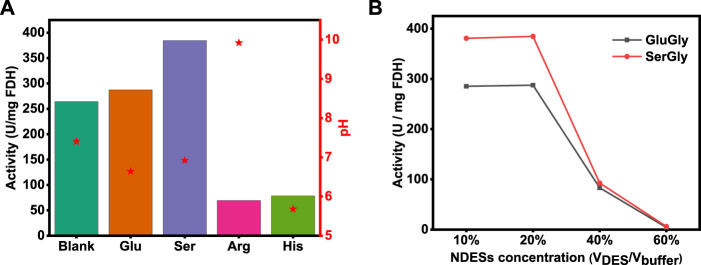
**(A)** FDH activity in four NADESs-based solutions (20% v/v) and Tris-HCl buffer. **(B)** FDH activity at different concentrations of SerGly and GluGly.

According to the kinetics of the multi-enzymatic reaction (Luo et al., 2015), the first two reactions (CO_2_→HCOOH→HCHO) were rate-limiting steps, and the second reaction (HCOOH→HCHO) was regulated by the product concentration of the first reaction. Therefore, the first reaction (CO_2_→HCOOH) plays a key role in the whole reaction, as confirmed by a previous study (Zhang et al., 2021c). Besides, biocatalyst FDH plays a key role in driving the first reaction, and thus the activity of FDH was evaluated in four NADESs (20% v/v) as well as a conventional buffer as a blank for comparison. As shown in Figure 3, the activity of FDH was enhanced by SerGly and GluGly, and SerGly presented the better enhancement with 384.6 U/mg, while the activities of FDH in ArgGly and HisGly were seriously decreased to 69.4 and 78.4 U/mg, respectively. To figure out the reason, the pH values of different NADES-based solutions were examined, and those for SerGly and GluGly were close to the optimal pH of FDH (7.0), while the pHs of ArgGly-based and HisGly-based solution were 9.9 and 5.6, respectively. Therefore, the pH values that are far from the optimal value of FDH are probably the main reason for the activity decline. For the case that the pH values of NADESs are close to the optimal pH of FDH, the enhancement can be explained as follows. Previous results revealed that the usage of ILs will increase the rigidity and stability of FDH, and also the substrate (CO_2_) residence time in the activity site of FDH (Zhang et al., 2018). The longer residence time will give more chances to adjust the substrate in the right position and thus make it more productive. If the enzyme is in an unfavorable media solution, the enzyme will not perform well with their unnormally folding conformation, leading to the decrease of the activity or even lose activity. Within the studied NADESs, SerGly with desirable pH exhibits the best enhancement on the enzyme activity.

To further identify the optimal NADESs concentration, the concentrations of SerGly and GluGly were set in the range from 10 to 60% (V_NADES_/V_buffer_) and evaluated their impact. As shown in Figure 3, the enzyme activity was enhanced and kept well in the concentration of NADESs from 10 to 20%, because the rigidity and stability of FDH were increased with increasing concentration from 1 to 20%, as described above. Aqueous solutions (water-rich IL mixtures) have also been confirmed to be the best media for proteins, being consistent with experimental results in this study (Kohno and Ohno, 2012). With further increasing the NADESs concentration from 20%, the enzyme activity was sharply decreased, which was probably attributed to the break of electrostatic balance in proteins by the charge of DES when subjected to high salt (DES) concentrations (Weingartner et al., 2012). Once the electrostatic balance in proteins is broken, the hydrophobic interaction plays a main role, resulting in aggregation of enzyme and thus decreasing the active sites of enzyme exposure out. Therefore, a high concentration of NADES will decrease the enzyme activity. Above all, SerGly is the best among the developed NADESs and 20% is optimal concentration. Unless specific statement, 20% NADES-based aqueous solution was used in the following study.

### Electrochemical Regeneration of Cofactor

**FIGURE 4:**
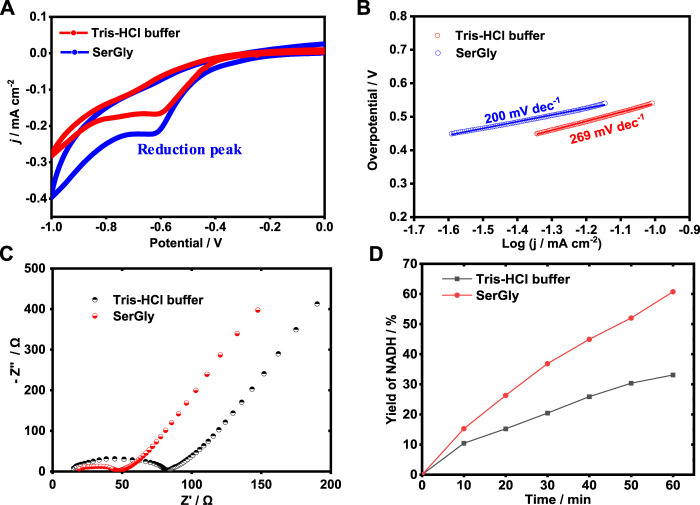
**(A)** Cyclic voltammogram (CV) curves of electrocatalyst in the SerGly-contained buffer and Tris-HCl buffer. **(B)** Tafel plot of electrocatalysts in the SerGly-contained buffer and Tris-HCl buffer. **(C)** Nyquist plots for electrocatalyst in the SerGly-contained buffer and Tris-HCl buffer. **(D)** Electro-reduction of NAD+ to NADH in the SerGly-contained buffer and Tris-HCl buffer.

The high performance of reducing NAD^+^ to NADH is crucial to achieving enzymatic conversion of CO_2_ in a sustainable way. Electrochemical regeneration of NADH was used in this study. In this part, the effect of buffers, i.e., DES-contained buffer (SerGly) and the conventional buffer (Tris-HCl), were investigated in the electrocatalysis and explore how the DES could boost the electrochemical reduction of NAD^+^ to NADH. First, the electrocatalytic activities of electrocatalyst (Rh complex) in SerGly-contained solution and Tris-HCl solution were separately characterized by cyclic voltammetry (CV). As shown in Figure 4A, within the cathodic potential from −1 to 0 V, a reduction peak, was observed at −0.62 V, indicating the reduction of Rh^III^ to Rh^I^, where the formed Rh^I^ can efficiently reduce NAD^+^ to NADH (Chen et al., 2019). At this reduction peak potential, the corresponding current density of SerGly-contained solution (−0.225 mA/cm^2^) is higher than that of conventional solution of −0.166 mA/cm^2^, confirming a higher electron transfer and NAD^+^ reduction efficiency in the SerGly-contained solution.

Next, the Tafel plot was separately examined in both solutions, as an indicator to quantify how easy/difficult to achieve a reaction, and the lower the overpotential, the easier the reaction (Kang et al., 2020). As presented in Figure 4B, the Tafel slope in SerGly-contained solution (200 dec^−1^) was lower than that in Tris-HCl buffer, indicating a lower overpotential under the given current density. This indicates a lower energy barrier in the SerGly-contained solution with a higher driving force. As reported by Faggion et al. report (Faggion et al., 2019), ILs could significantly reduce the overpotential in the electrochemical reduction of CO_2_ and improve the electrochemical efficiency. This observation is consistent with our findings here.

To reveal the difficulty of kinetic reaction, the interface between electrode and electrolyte was investigated by electrochemical impedance spectroscopy (EIS). Figure 4C exhibits typical results of impedance spectra (Nyquist plots), where Z’ and Z” are the real variable and the negatively imaginary variable of impedance, respectively. A semi-circular part at high frequencies in Figure 4C corresponds to the electron-transfer limited process (the electrode/solution interface), and a linear part at low frequencies corresponds to the diffusion process. Charge transfer resistance (R_ct_) in the SerGly-contained solution was estimated to be 48 Ω, which is remarkably lower than that in the Tris-HCl buffer of 82 Ω. The results with lower R_ct_ in the SerGly-contained solution suggested the faster interfacial charge transfer rate between the electrolyte (SerGly) and the working electrode, which is superior to the Tris-HCl buffer in electrocatalysis principally.

To verify the enhancement of NAD^+^ reduction by NADESs, the electrochemical reduction of NAD^+^ to NADH was separately conducted in the SerGly-contained solution and Tris-HCl solution. As shown in Figure 4D, the yield of NADH in the SerGly-contained solution was 60% in 1 h, which was approximately two times higher than that in Tris-HCl buffer. The higher reduction rate of NAD^+^ to NADH in SerGly-contained solution was attributed to higher electron transfer by SerGly, which has been demonstrated by the electrochemical characterization above. Therefore, besides serving as a co-solvent to enhance the enzyme activity, SerGly can also boost electrochemical conversion and is a desirable co-solvent for the enzymatic reaction.

### Multi-Enzymatic Reaction for Methanol Production

**FIGURE 5:**
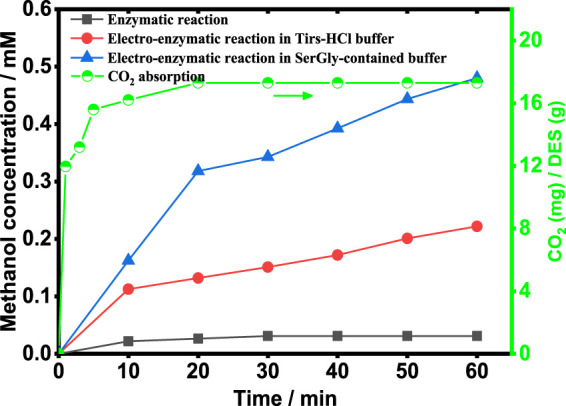
Electro-enzymatic reaction for methanol production under different conditions and the CO_2_ concentration in the SerGly-contained solution.

To establish a sustainable process to produce methanol and enhance the process by NADES, the enzymatic reaction coupled with NADH electro-regeneration in the SerGly-contained buffer and Tris-HCl buffer was implemented, and the control reaction (the enzymatic reaction without NADH regeneration) was also studied for comparison. Therefore, three systems were designed and evaluated, as shown in Figure 5. The enzymatic reaction without NADH electro-regeneration quickly reached equilibrium with low methanol concentration (0.03 mM), since such multi-enzymatic reaction is reversible and CO_2_ conversion was inhibited by the accumulation of byproduct NAD^+^ over time (Luo et al., 2015). Applying with electro-reduction of NAD^+^ to NADH, the methanol concentration was 0.22 mM, significantly increased up to 7 times higher than the control reaction. It indicated that the byproduct NAD^+^ was efficiently reduced to cofactor NADH, achieving the removal of NAD^+^ and the increased NADH concentration. To further boost the electro-enzymatic conversion of CO_2_, SerGly was used to enhance the whole process, i.e., remarkably increase the CO_2_ conversion. As a result, the rate of methanol generation reached 0.48 mM/h, which is two times higher than that in the Tris-HCl buffer (0.22 mM/h) and 16 times higher than the control reaction. Being consistent with the above investigation, SerGly enhanced not only enzyme activity but also NADH electro-regeneration rate, which was probably the main reason to improve the CO_2_ conversion. Besides, SerGly increased the CO_2_ concentration in the solution. As shown in Figure 5, the CO_2_ solubility in the SerGly-contained solution was 17.3 mg-CO_2_/g-DES at room temperature, which is 11 times higher than that in the Tris-HCl solution (1.5 mg-CO_2_/g-water). Zhang et al. (2021c) demonstrated that increasing the CO_2_ concentration could significantly drive the reversible reaction forward and thus increase the CO_2_ conversion in the enzymatic reaction. Therefore, SerGly plays a role in enhancing enzyme activity and cofactor electro-regeneration as well as in the enrichment of the substrate CO_2_, and finally enhances the process of the electro-enzymatic conversion of CO_2_.

## Conclusion

In this study, four NADESs, GluGly, SerGly, HisGly, and ArgGly, were developed and characterized for the first time. The performance of these NADESs was evaluated in the enzymatic reaction, in which SerGly presents the best results on the enzyme activity. The SerGly-based solution was further investigated in the electro-enzymatic conversion of CO_2_. It was found that 1) the CO_2_ solubility in the SerGly-based solution is 11 times higher than that in the conventional buffer, contributing to the enhancement of the enzymatic CO_2_ conversion; 2) NADH electro-regeneration rate was approximately 2 times higher than that in the conventional buffer, providing the fundamental condition to accelerate the CO_2_ conversion, and 3) the methanol production from the multi-enzymatic conversion of CO_2_ coupled with the NADH electro-regeneration is 2 times higher than that in the Tris-HCl buffer (0.22) and 16 times higher than the control reaction. This work developed a novel NADES with desirable biocompatibility, high electrocatalytic performance, and high CO_2_ solubility, enhancing the whole process of electro-enzymatic conversion of CO_2_ and being promising for the enzymatic catalysis, electrocatalysis, and CO_2_ capture research field.

## Data Availability

The original contributions presented in the study are included in the article/Supplementary Material, further inquiries can be directed to the corresponding authors.
