# Supplementary material for: Natural Deep Eutectic Solvents Enhanced Electro-Enzymatic Conversion of CO2 to Methanol
Source: Front Chem. 2022 May 27;10:894106. doi: 10.3389/fchem.2022.894106 (PMC9184674; doi:10.3389/fchem.2022.894106)
Supplement: Supplementary file 1 [file DataSheet1.PDF]

Natural deep eutectic solvents enhanced electro-enzymatic conversion of CO_2 to methanol

Zhibo Zhang,[1]* Hui Wang,[2] Yi Nie,[3,4] Xiangping Zhang,[4] Suojiang Zhang,[4] Xiaoyan Ji [1]*

[1]Energy Engineering, Division of Energy Science, Luleå University of Technology, 97187, Luleå, Sweden;

[2]State Key Laboratory of Materials-Oriented Chemical Engineering, Nanjing Tech University, Nanjing 210009, P. R. China

[3]Zhengzhou Institute of Emerging Industrial Technology, Zhengzhou 450000, Henan Province, China;

[4]Beijing Key Laboratory of Ionic Liquids Clean Process, CAS Key Laboratory of Green Process and Engineering, State Key Laboratory of Multiphase Complex Systems, Institute of Process Engineering, Chinese Academy of Sciences, Beijing, 100190, China.

Corresponding Author: Zhibo Zhang, Email: zhibo.zhang@ltu.se

Corresponding Author: Xiaoyan Ji, Email: xiaoyan.ji@ltu.se

Table S1. The density and viscosity of NDESs at different temperatures.

Temperature	Density (g/cm^3)				Viscosity (mPa·s)			
(°C)	GluGly	SerGly	ArgGly	HisGly	GluGly	SerGly	ArgGly	HisGly
20	1.261	1.263	1.275	1.263	1550	1588.2	1501.1	1720.8
30	1.255	1.256	1.270	1.257	638.1	668.6	549.7	711.9
40	1.249	1.250	1.264	1.251	300.0	311.4	218.0	328.8
50	1.242	1.244	1.258	1.245	155.5	160.2	96.2	168.1
60	1.236	1.237	1.252	1.238	87.1	89.6	47.7	93.0
70	1.229	1.231	1.246	1.231	52.8	54.4	25.7	55.6
80	1.223	1.224	1.240	1.225	33.8	34.8	14.6	35.3

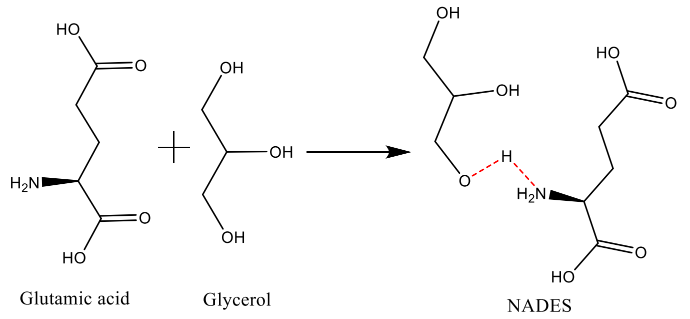

Figure S1. Proposed reaction between HBA and HBA (GluGly).

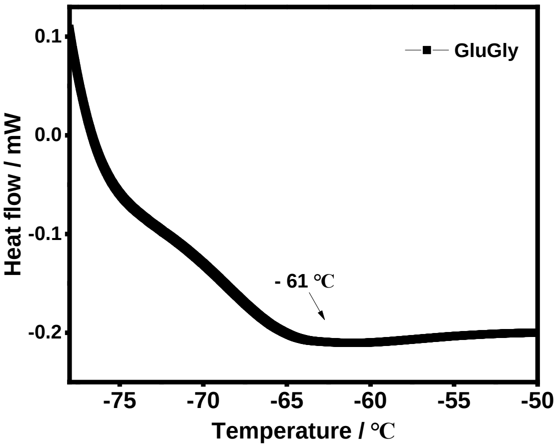

Figure S2. DSC curve of GluGly.
